# Plasma Glutamine Levels in Relation to Intensive Care Unit Patient Outcome

**DOI:** 10.3390/nu12020402

**Published:** 2020-02-03

**Authors:** Renée Blaauw, Daan G. Nel, Gunter K. Schleicher

**Affiliations:** 1 Division of Human Nutrition, Faculty of Medicine and Health Sciences, Stellenbosch University, Cape Town 8000, South Africa; 2 Centre for Statistical Consultation, Stellenbosch University, Cape Town 8000, South Africa; dgnel@sun.ac.za; 3 Wits Donald Gordon Medical Centre ICU, Johannesburg 2193, South Africa; schleicher@worldonline.co.za

**Keywords:** plasma glutamine, clinical outcome, critically ill adult patients

## Abstract

Low and high plasma glutamine levels are associated with increased mortality. This study aimed to measure glutamine levels in critically ill patients admitted to the intensive care unit (ICU), correlate the glutamine values with clinical outcomes, and identify proxy indicators of abnormal glutamine levels. Patients were enrolled from three ICUs in South Africa, provided they met the inclusion criteria. Clinical and biochemical data were collected. Plasma glutamine was categorized as low (<420 µmol/L), normal (420–700 µmol/L), or high (>700 µmol/L). Three hundred and thirty patients (median age 46.8 years, 56.4% male) were enrolled (median APACHE II score) 18.0 and SOFA) score 7.0). On admission, 58.5% had low (median 299.5 µmol/L) and 14.2% high (median 898.9 µmol/L) plasma glutamine levels. Patients with a diagnosis of polytrauma and sepsis on ICU admission presented with the lowest, and those with liver failure had the highest glutamine levels. Admission low plasma glutamine was associated with higher APACHE II scores (*p* = 0.003), SOFA scores (*p* = 0.003), C-reactive protein (CRP) values (*p* < 0.001), serum urea (*p* = 0.008), and serum creatinine (*p* = 0.023) and lower serum albumin (*p* < 0.001). Low plasma glutamine was also associated with requiring mechanical ventilation and receiving nutritional support. However, it was not significantly associated with length of stay or mortality. ROC curve analysis revealed a CRP threshold value of 87.9 mg/L to be indicative of low plasma glutamine levels (area under the curve (AUC) 0.7, *p* < 0.001). Fifty-nine percent of ICU patients had low plasma glutamine on admission, with significant differences found between diagnostic groupings. Markers of infection and disease severity were significant indicators of low plasma glutamine.

## 1. Introduction

Immunonutrition refers to the administration of nutrients to modulate the immune system to improve the clinical outcome. These nutrients include glutamine, arginine, omega-3 fatty acids, and a host of antioxidants [1]. Of these, glutamine is the most studied supplement. Numerous meta-analyses have reported on several systemic and clinical benefits attributed to glutamine supplementation, including decreased systemic infections [2,3,4,5,6,7,8,9], decreased intensive care unit (ICU) and hospital stay [2,4,5,6,7,9,10], decreased cost [11], and ultimately decreased mortality [3,6,7,10]. However, not all studies have been positive, and evidence of harm has also been reported [12,13]. It should be noted that the former two studies did not supplement glutamine according to the recommended indications and added antioxidants and other immunonutrients.

The results of these trials clearly demonstrated that glutamine supplementation is not without risk and that there should be clearer guidelines on which patients would benefit from glutamine supplementation, what is the suitable dosage, route, and duration of administration. In their publication addressing the question “Where did we go wrong in our pursuit of knowledge regarding the role of glutamine in the ICU?”, the authors of the REDOXS (Reducing deaths due to oxidative stress) trial acknowledged that they assumed that all critically ill patients had low plasma glutamine levels and would therefore benefit from high-dose supplementation [14]. More recent work on this topic has questioned this assumption. In human subjects, a circulating plasma glutamine level above 420 µmol/L is regarded as normal [15]. Low plasma glutamine levels have been associated with increased mortality [15,16,17,18], and similarly, high plasma levels (above 700 µmol/L) have also been associated with increased mortality [15,16,19]. Thus, plasma glutamine levels may have a U-shaped curve in their clinical response and effect on mortality [15,16]. As circulating plasma glutamine levels are not routinely measured in most hospitals, it becomes important to identify potential proxy indicators of abnormal glutamine levels. The latter could assist with the clinical decision on when to provide and when to withhold glutamine supplementation.

The primary aim of this study was to establish baseline glutamine levels in critically ill patients on admission to ICU with various clinical conditions and to correlate the values with clinical outcomes and mortality. The secondary aim was to identify surrogate clinical and biochemical markers of glutamine deficiency or elevated plasma glutamine levels.

## 2. Materials and Methods

### 2.1. Subjects and Study Design

This multi-center cohort study enrolled adult patients (>18 years old) admitted to the surgical and medical ICU’s of 3 Hospitals in South Africa (Tygerberg Hospital, Cape Town; Wits Donald Gordon Medical Centre (WDGMC), Johannesburg and Kimberley Hospital Complex, Kimberley) during the period July 2016 until December 2018. Inclusion criteria were an expected ICU stay of ≥3 days and no glutamine supplementation during the previous 14 days before admission

The sample size of 300 subjects was calculated based on the primary aim to determine the proportion of patients with glutamine deficiency with a precision of 9%, 90% power, and a 95% confidence interval. For sub-group analysis (e.g., glutamine deficient versus glutamine sufficient), a minimum of 135 subjects were needed per group, using 90% power and 95% confidence interval.

In each of the three hospitals, subjects were stratified by disease category (medical versus surgical). A minimum of 50 surgical and 50 medical ICU subjects were selected per hospital. The first 50 surgical and 50 medical subjects that met the inclusion criteria per facility were approached for participation.

### 2.2. Measurements

Baseline data (as discussed below) were determined within 24 h of ICU admission and repeated on day 7 (or on ICU discharge if discharged between day 3 and 7).

### 2.3. Anthropometric Measurements

Weight and height were recorded on admission to the ICU and weight measurement was repeated weekly for the duration of hospital stay. Body mass index (BMI) (weight (kg)/height (m^2^)) was calculated and interpreted according to acknowledged guidelines [20].

### 2.4. Clinical and Medical Parameters

Routine clinical information was recorded, including length of stay in the ICU and hospital, medical/surgical diagnosis, disease severity indicators e.g., Acute Physiology, Age, Chronic Health Evaluation Score (APACHE II) (score (on admission) and Sequential Organ Failure Assessment (SOFA) score (on admission and on day 7), need for mechanical ventilation (during ICU stay), development of complications that required medical intervention anytime during the period of hospitalization, and mortality (during ICU stay and hospitalization).

### 2.5. Dietary Intake

Nutritional support was provided to all subjects using standard operating protocols for each hospital. Daily nutritional intake of subjects was recorded in detail, including glutamine content of feeds and supplements.

### 2.6. Plasma Glutamine

For the determination of plasma glutamine levels, a 10 mL blood sample was taken at ICU admission and again at day 7 (or on the day of discharge if earlier than day 7). All samples were centrifuged within 30 min and stored at −80 °C until analyses, using Liquid Chromatography Mass Spectrometry. This analysis was done at the Central Analytical Facilities of Stellenbosch University. Plasma glutamine serum levels were categorized as low (<420 µmol/L), normal (420–700 µmol/L), and high (>700 µmol/L) [15].

### 2.7. Data Analysis

Microsoft Excel (Microsoft, Redmond, WA, USA) was used to capture the data and STATISTICA (version 13.4, 2018; StatSoft, Tulsa, OK, USA) was used to analyze the data.

For continuous normally distributed variables, means and standard deviations were used as measures of central location and spread respectively and for ordinal or non-normal data medians and inter-quartile ranges (IQR). Relationships between two continuous variables were analyzed with regression analysis and the strength of the relationship measured with the Pearson correlation or with Spearman correlation for non-normal or ordinal variables. The relationships between continuous response variables and nominal input variables were analyzed using one-way analysis of variance (ANOVA) for completely randomized designs or the Kruskal–Wallis test if the residuals were not normally distributed. For repeated measures with k repetitions, repeated-measures ANOVA was used with the compound symmetry assumption on correlations over time. For k = 2, paired *t*-tests were used or Wilcoxon tests for non-normal data. Receiver-operating characteristic (ROC) curves were used to determine the optimal cut-off value for continuous responses versus dichotomous nominal variables. Nominal variables were compared using contingency tables and the likelihood ratio chi-square test. A *p*-value less than 5% indicated statistical significance in hypothesis testing. Missing data were not replaced or imputed.

### 2.8. Ethical Principles

The study was approved by the Health Research Ethics Committees, Stellenbosch University and University of the Witwatersrand (N14/08/114 and M170981, respectively). We also received approval and permission to conduct the study from all three hospitals.

The study was carried out following the rules of the Declaration of Helsinki of 1975, revised in 2013. All subjects provided voluntary informed consent before participation in the study and could withdraw at any time.

## 3. Results

A total of 330 subjects, 56.4% males, with a median age of 46.8 (IQR: 32.0–60.2) years participated. (Table 1).

### 3.1. Medical Data

An even distribution was found between medical and surgical diagnostic groups. Primary diagnosis of sepsis dominated (35.8%), followed by polytrauma (18.8%). A relatively small percentage of subjects were HIV positive (18.0% of those for whom the status was available), with an even smaller group with microbiologically proven tuberculosis (5.0% of those for whom the status was available). The majority were mechanically ventilated (67.2%) (Table 1).

On admission, the median APACHE II score was 18.0 and the median SOFA score was 7.0. Day 7 median SOFA score was 3.0 (IQR: 1.0–7.0). The total duration of ICU stay was half that of hospital stay (7.0 versus 14.0 days respectively). (Table 1). A quarter of all subjects died during the study (*n* = 78/316, 24.6%), and 54 of the deaths occurred during the first seven days.

### 3.2. Anthropometry

A median BMI of 25.7 kg/m^2^ was reported for the group (IQR: 22.9–29.9 kg/m^2^). Underweight was present in 4.3% and overweight/obesity (BMI ≥ 25 kg/m^2^) in 57.3% of subjects. (Table 1).

### 3.3. Dietary Intake

The majority of subjects (*n* = 251, 76.1%) received nutritional support during the first seven days. Of those, enteral nutritional supplements formed the largest group (*n* = 195, 77.7%), followed by combination therapy of enteral and parenteral nutrition (*n* = 29, 11.6%) and parenteral nutrition only (*n* = 27, 10.8%). For those receiving nutrition support, the mean energy intake during the first seven days of ICU stay in ICU was 941.0 ± 688.3 kcal (median 1027.3 kcal, IQR: 212.8–1484.9 kcal). This amounted to a mean of 12.6 ± 9.5 kcal/kg body weight per day. The mean protein intake was 44.2 ± 33.2 g (median 46.3 g, IQR: 10.7–70.1 g), amounting to 0.59 ± 0.5 g/kg body weight per day. It should be noted that some subjects also received fluid diets and oral nutritional supplements. However, because the intake of these fluids was inconsistent and not well charted, we did not consider this in our calculations. A total of 45 subjects received glutamine as part of the parenteral nutrition support. Taken as an average intake for all subjects receiving nutrition support, this was calculated as 1.23 ± 3.8 g per day (0.015 ± 0.05 g/kg body weight per day).

### 3.4. Biochemical Data

Even though an improvement was noted for most parameters, all values remained abnormal by day 7 (Table 2).

### 3.5. Plasma Glutamine

**Figure 1:**
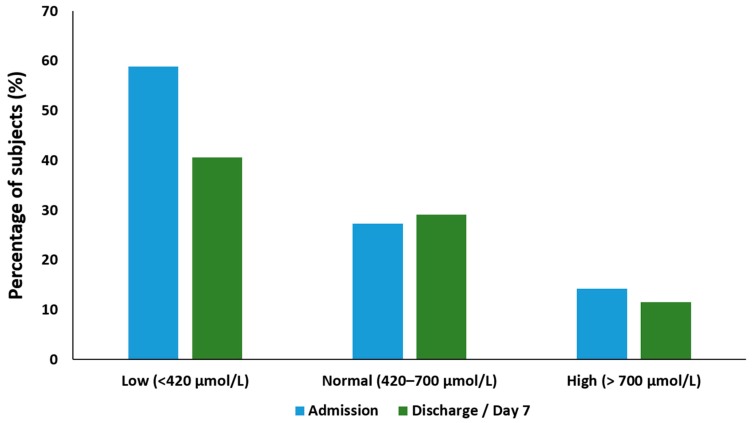
Plasma glutamine categories for admission and day 7.

On admission, the mean plasma glutamine concentration was 464.9 ± 344.1 µmol/L (median 382.2, IQR: 280.7–572.2 µmol/L). More than half of the subjects (*n* = 193/330, 58.5%) had low plasma glutamine levels (<420 µmol/L). High plasma glutamine (>700 µmol/L) was found in 14.2% (*n* = 47/330) of subjects. The mean plasma glutamine on day 7 was 509.8 ± 543.9 µmol/L (median 418.9, IQR: 323.7–562.2 µmol/L). Expressed as a percentage of those that entered the study, 40.6% (*n* = 134/330) had low plasma glutamine levels on day 7, while 11.5% (*n* = 38/330) fell in the high plasma glutamine category. The proportion of subjects in the normal glutamine category increased slightly from 27.3% (*n* = 90/330) at admission to 29.1% (*n* = 96/330) on day 7. (Figure 1). No discharge (day 7) glutamine samples were obtained for 18.8% (*n* = 62/330) of subjects.

**Figure 2:**
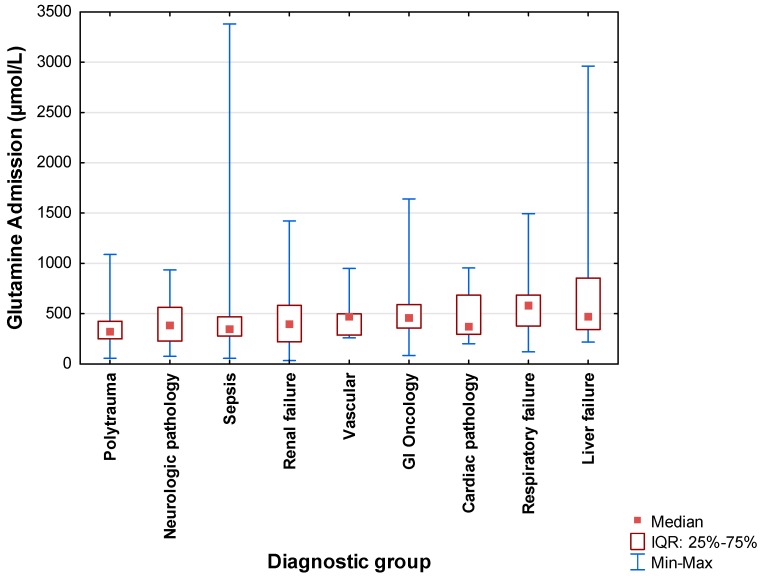
Boxplots indicating plasma glutamine levels per diagnostic group. GI—Gastrointestinal.

The admission plasma glutamine concentrations for each of the primary diagnostic categories are displayed in Figure 2 by means of boxplots. The lowest median values were recorded for the polytrauma group (326.8 µmol/L; IQR: 249.8–424.7 µmol/L), followed by sepsis (351.8 µmol/L; IQR: 278.28–467.5 µmol/L). Significant differences were found between polytrauma with respiratory failure (*p* = 0.028) and liver failure (*p* = 0.024, Kruskal–Wallis tests).

More than half of subjects with polytrauma (74.2%), sepsis (69.5%), cardiac pathology (55.6%), neurologic pathology (55.0%), and renal failure (54.2%) presented with low plasma glutamine on admission. On the other hand, 28.9% of subjects with liver failure and 25.0% of those with respiratory failure presented with a high plasma glutamine value.

### 3.6. Changes in Glutamine Status from Admission to Discharge

A paired *t*-test was used to determine the difference in mean glutamine concentrations (day 7/discharge (509.8 ± 543.9) − admission (466.9 ± 333.9 µmol/L)) (*n* = 268). A difference of 42.9 ± 542.7 µmol/L (*p* = 0.211) indicated a slight increase in circulating glutamine levels over the first seven days. When excluding the 45 subjects that received parenteral nutrition (PN) glutamine supplementation, the mean glutamine difference over time was 17.02 µmol/L (*p* = 0.505).

### 3.7. Associations between Plasma Glutamine and Various Clinical Outcomes

Baseline plasma glutamine were weakly negatively correlated with CRP on admission (r = −0.287, *p* < 0.0001), serum urea on admission (r = −0.124, *p* = 0.026), and weakly positively correlated with total bilirubin on admission (r = 0.262, *p* < 0.001), serum ALT on admission (r = 0.119, *p* = 0.032), and SOFA day 7 (r = 0.133, *p* = 0.024). (Spearman correlations).

Table 3 and Table 4 compare subjects with a low admission glutamine (<420 µmol/L) and those with a normal or elevated glutamine level (≥420 µmol/L) in terms of clinical and biochemical outcomes. Subjects with low glutamine on admission were more likely to have a positive tuberculosis status (Chi^2^ = 4.75, *p* = 0.029), be on mechanical ventilation (Chi^2^ = 12.65, *p* < 0.001), and require nutritional support (Chi^2^ = 5.74, *p* = 0.016). A low admission plasma glutamine (<420 µmol/L) was also significantly associated with higher APACHE II scores (*p* = 0.003), higher SOFA scores on admission (*p* = 0.003), higher CRP values on admission (*p* < 0.001) and day 7 (*p* = 0.002), higher serum urea on admission (*p* = 0.008) and day 7 (*p* = 0.028), and higher serum creatinine on admission (*p* = 0.023) and day 7 (*p* = 0.006). It was also associated with lower serum albumin on admission (*p* < 0.001).

This study did not find significant associations between admission plasma glutamine and length of stay (ICU or hospital), number of complications, or mortality during the hospitalization period.

**Figure 3:**
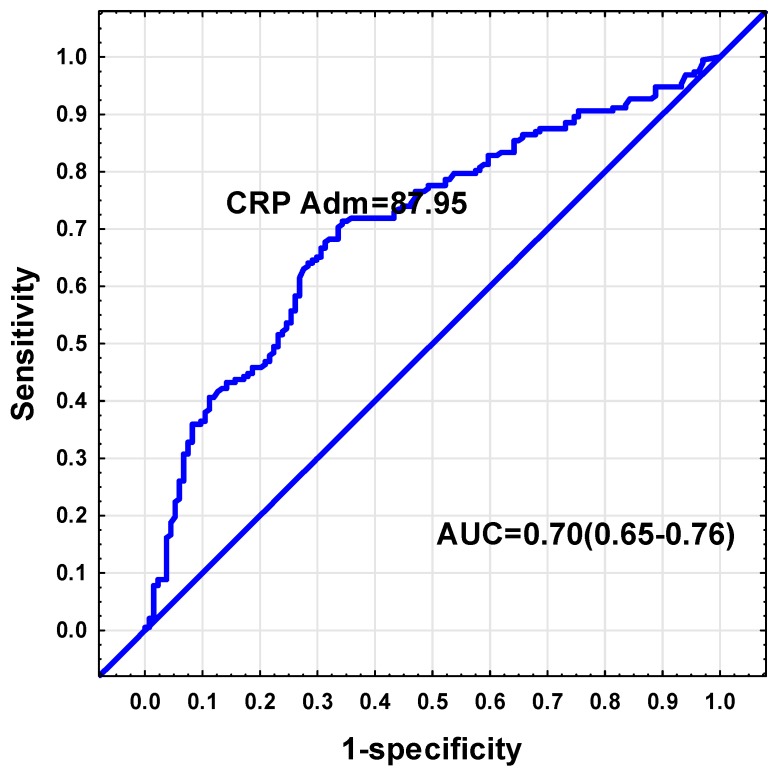
Receiver-operating characteristic (ROC) curve indicator of low plasma glutamine.

Receiver-operating characteristic (ROC) curves were used to determine which parameters (admission APACHE II, SOFA, CRP, albumin, urea, creatinine, total bilirubin, AST, ALT, LDH, and SOFA day 7) could be used to discriminate between low and sufficient admission glutamine levels. From these parameters, the only analysis with an area under the curve ≥0.7, was found for CRP. The ROC curve analysis indicated a CRP threshold value of 87.95 mg/dL to be indicative of low plasma glutamine (Sensitivity = 71%, Specificity = 66%, positive predictive value = 75%, negative predictive value = 62%, AUC = 0.70, 95% CI: 0.65–0.76, *p* < 0.001). (Figure 3). Of those subjects with a CRP value exceeding 87.95 mg/dL (*n* = 179), 75.9% had plasma glutamine levels below 420 µmol/L.

A small proportion of subjects (*n* = 47, 14.2%) fell in the high serum glutamine category on admission. The mean glutamine concentration for this group was 1067.9 ± 521.2 µmol/L (median 898.9 µmol/L and IQR: 815.8–1088.3 µmol/L). Subjects with high glutamine levels were more likely to be in the medical ward (Chi^2^ = 4.40, *p* = 0.036); had a significantly shorter hospital length of stay (*p* = 0.04); and presented with significantly lower CRP values (*p* = 0.003). No significant associations were found between high glutamine levels on admission and indicators of illness severity (SOFA and APACHE II scores) or mortality. However, due to the small number of subjects in the high glutamine category, the power to detect these associations was weak.

## 4. Discussion

This study aimed to determine the circulating plasma glutamine levels of critically ill subjects on admission to three ICUs in South Africa. The average glutamine concentration was 464.9 µmol/L, ranging from a minimum of 34.2 to a maximum level of 3380 µmol/L. More than half (59%) of the subjects had glutamine deficiency and this correlated significantly with biochemical markers of infection/inflammation (CRP level) and illness severity (SOFA and APACHE II scores).

The average plasma glutamine level in this study compares well to those reported in the literature of similar patient groups, ranging from an average level of 420–497 µmol/L [15,16,17,21,22]. The average age of the subjects was 47 years, which is similar to another South African study [22], but younger than most other studies, which reported average ages of 59 years [15], 64 years [16], 68 years [21], and 74 years [17]. The majority of subjects had a primary ICU admission diagnosis of sepsis, followed by polytrauma and gastrointestinal oncology cases. The overall study mortality was 24%, with most of the deaths occurring in the ICU.

Although patients did not receive routine glutamine supplementation during their ICU admission, a small number of subjects received parenteral feeds containing glutamine at the discretion of the treating physician. On average, we estimated that the glutamine supplements only amounted to 0.015 g/kg glutamine per day, which is unlikely to have affected the results of this observational study.

The prevalence of low plasma glutamine was 59% at baseline, and by day 7, 50% of subjects still had a low plasma glutamine level. Using similar cut-off values to define glutamine deficiency, our results are consistent with some of the highest reported figures. The prevalence of glutamine deficiency in studies from The Netherlands ranged between 65% [23], 55% [21], and 31% [17]; a Swedish study reported 44% deficiency [15]; a study from Japan reported a prevalence of 33% [16] and from South Africa a prevalence of 38% was reported [22].

Significant clinical differences were found in circulating plasma glutamine levels according to diagnostic category, with polytrauma subjects having the lowest levels, followed by patients with sepsis. Those with liver failure had the highest mean glutamine values. Oudemans-van Straaten et al. [17] also reported low glutamine levels with a narrow range for the sepsis group, although the patients with sepsis in their study also had some outliers. Other groups have also reported on liver failure patients having elevated glutamine levels [16], with Helling et al. [24] reporting a correlation between plasma glutamine levels and severity of liver disease. The former group proposed that liver glutamine uptake is reduced in cases of liver damage, resulting in raised plasma levels [16].

A low baseline glutamine level was associated with biochemical markers of sepsis (higher CRP and lower albumin values) and injury severity (higher APACHE II and SOFA scores). Contradictory findings are reported in the literature with injury severity indicators not strongly associated with circulating glutamine in some cases [15,16] and positively associated in other cases [23]. The correlation with markers of infection and inflammation has been confirmed by others, with lower plasma glutamine levels found to be associated with lower albumin levels [17,18], higher CRP levels [18,22], and higher circulating IL-1β and IL-6 levels [18]. In some studies, low glutamine levels were also associated with renal impairment, but this was not confirmed in other studies [16]. Based on the results of the REDOXS trial, harmful outcomes were reported in the subgroup of patients with baseline renal dysfunction who received glutamine or antioxidant supplements [25]. This suggests the potential danger of routine glutamine supplementation in patients with renal dysfunction without prior assessment of plasma levels. Although it was not reported what proportion of this subgroup of patients in the REDOXS study had glutamine deficiency at baseline, 31% of the total study population had glutamine deficiency and 15% had supra-normal levels. Plasma glutamine levels were only tested on a subgroup of the overall study population (66 of the total 1223) [12]. In our study, biochemical indicators of hepatic dysfunction (raised bilirubin, AST, and ALT levels) were associated with elevated serum glutamine levels. Similar findings have been reported by others [16,24].

We noted that patients with a low baseline glutamine had a higher ICU mortality, but this association was not significant. Again, contradictory findings are reported in the literature, with no strong link between glutamine levels and mortality [26], mortality associated with low baseline glutamine levels [17,18], or mortality associated with either low or high glutamine levels [15,16].

Using the predictors of a low plasma glutamine, we determined cut-off values to differentiate between a low and sufficient glutamine status. A CRP value exceeding 88 mg/dL was associated with a low plasma glutamine status. This CRP value is similar to a previous study, which found a cut-off CRP of 95.5 mg/dL (Specificity = 69%, Sensitivity = 62.2%, AUC = 0.759, 95% CI: 0.653–0.865), *p* < 0.0001) [22]. We reported that two-thirds of subjects with CRP values exceeding this cut-off had low plasma glutamine values, which can be regarded as a reasonably good indicator of inadequate levels.

This study did not set out to assess the effect of glutamine supplementation on clinical outcomes and therefore we cannot comment on supplementation regimes. We describe the glutamine status of subjects on admission to and on day 7 of ICU stay and have determined associated clinical markers of deficiency and subsequent outcomes. We found significant differences in circulating glutamine levels between ICU admission diagnostic categories, ranging from deficient to supra-normal levels. This highlights the danger of routine glutamine supplementation and strengthens the importance of applying clinical judgement to every patient. Recently, the accuracy of a point-of-care instrument to screen for hypoglutaminemia and hyperglutaminemia was confirmed [27]. Routine use of this device might assist with clinical decision-making at the bedside. During periods of stress, glutamine production may not be sufficient to keep up with demand [28,29] and during periods of glutamine supplementation, endogenous glutamine production does not decrease [30,31]. This is associated with the observation that glutamine deficiency is common in ICU patients, but also that routine glutamine supplementation in ICU patients without documented clinical deficiency may result in harmful outcomes [25]. For this reason, *routine* supplementation of glutamine to all critically ill patients is not recommended by various Societies like ESPEN (European Society for Clinical Nutrition and Metabolism) [32]; ASPEN (American Society for Parenteral and Enteral Nutrition) [33]; and the German Nutrition Guidelines [34]. Although glutamine is thought to have an important role in critically ill patients, the recognized contra-indications to glutamine supplementation must be adhered to, such as the presence of multiple organ failure (especially renal and liver failure) [1,7,10,14,32,34] and patients with septic shock requiring vasopressor support [1,7,10,34]. When supplementing with glutamine, the dose should not exceed 0.5 g/kg body weight per day [7,10,14]; it should not be supplemented during the early acute phase of critical illness [10,34]; it should be administered together with full nutrition support [14] and the glutamine dose should not exceed 30% of the prescribed nitrogen supply [7].

## 5. Conclusions

Nearly two-thirds (59%) of all critically ill medical and surgical subjects admitted to three South African ICUs had a low plasma glutamine level on admission to ICU, and 14% had elevated glutamine levels. Within each of the diagnostic categories, a range of low to high plasma glutamine values were found. However, patients admitted with the diagnosis of polytrauma and sepsis had the lowest glutamine values. Conversely, patients admitted with liver failure had the highest plasma glutamine values. Biochemical markers of infection and inflammation (elevated CRP and decreased serum albumin levels) and clinical scores of disease severity (elevated APACHE II and SOFA scores) were significant indicators of low plasma glutamine levels. Even though these markers of disease severity correlated significantly with low plasma glutamine levels, the latter were not associated with all observed clinical outcomes (such as length of stay or mortality) apart from the need for mechanical ventilation. These biochemical and clinical risk factors may help identify ICU patients at highest risk for glutamine deficiency. The reasons for these low levels are complex and multifactorial, including underlying pathology, inflammation, sepsis, nutritional state, and malignancy.

The data were gathered in three ICUs in South Africa that represent three different geographical regions and included state and private institutions. We believe that it provides a good representation of the patient profile in our country. The results are clinically relevant as they highlight the diagnostic groupings most associated with a low glutamine status. It also identifies measures of disease severity and clinical and biochemical markers that correlate significantly with hypoglutaminemia. Patients meeting these criteria are at highest risk for glutamine deficiency and should be further investigated with formal glutamine plasma levels. Future studies should investigate the effect of glutamine repletion on clinical outcomes within specific diagnostic groupings.

## Figures and Tables

**Table 1:** Demographic and clinical data on admission.

Variables	Unit	
	*n*	Percentage (%)
**Gender** (*n* = 330)		
Male	186	56.4
Female	144	43.6
**Disease category** (*n* = 330)		
Medical	147	47.6
Surgical	173	52.4
**Primary diagnosis** (*n* = 330)		
Sepsis	118	35.8
Polytrauma	62	18.8
Gastrointestinal oncology	42	12.7
Liver failure	28	8.5
Renal failure	24	7.3
Neurologic pathology	20	6.1
Respiratory failure	20	6.1
Cardiac pathology	9	2.7
Vascular	7	2.1
**Mechanical ventilation on admission** (*n* = 330)		
Yes	223	67.6
No	107	32.4
**HIV positive** (*n* = 250)		
Yes	45	18.0
No	205	82.0
**Tuberculosis positive** (*n* = 181)		
Yes	9	5.0
No	172	95.0
**BMI, categories** (kg/m^2^) (*n* = 328)		
Underweight (<18.5 kg/m^2^)	14	4.3
Normal weight (18.5–24.9 kg/m^2^)	126	38.4
Overweight (25.0–29.9 kg/m^2^)	106	32.3
Obese (30.0–39.9 kg/m^2^)	82	25
	**Mean ± SD**	**Median (IQR)**
**Age** (years)	47.4 ± 16.6	46.8 (32.0–60.2)
**Illness severity indicators**		
APACHE II score (Admission) (*n* = 330)	18.6 ± 8.6	18.0 (11.0–25.0)
SOFA score (Admission) (*n* = 330)	7.1 ± 3.8	7.0 (4.0–10.0)
**Length of stay (days)** (*n* = 330)		
ICU	9.5 ± 8.7	7.0 (4.0–11.0)
Hospital	18.6 ± 14.9	14.0 (8.0–26.0)

HIV: Human Immunodeficiency Virus; ICU: Intensive care unit; BMI: Body mass index; APACHE: Acute Physiology, Age, Chronic Health Evaluation Score; SOFA: Sequential organ Failure Assessment Score; SD: Standard deviation; IQR: Inter quartile range.

**Table 2:** Biochemical results at admission and discharge.

Biochemical Variable	Normal Range	Unit	Admission		Day 7/Discharge	
				*n*		*n*
Albumin (g/L)	35–52	Mean ± SD Median IQR	25.8 ± 7.7 26.0 21.0–31.0	318	25.0 ± 13.9 24.0 19.0–29.0	280
Urea (mmol/L)	2.6–7.0	Mean ± SD Median IQR	11.9 ± 16.6 6.9 4.5–14.6	324	12.7 ± 12.7 7.8 4.5–14.1	289
Creatinine (µmol/L)	60–100	Mean ± SD Median IQR	206.7 ± 290.4 103.0 68.0–197.5	324	167.5 ± 218.9 72.0 55.0–147.0	289
Total bilirubin (µmol/L)	0–21	Mean ± SD Median IQR	25.1 ± 47.1 11.0 7.0–3.0	322	23.2 ± 44.3 10.0 6.0–20.0	281
AST (u/L)	8–20	Mean ± SD Median IQR	289.9 ± 1105.7 53.0 28.0–153.0	321	84.3 ± 127.1 47.5 29.0–83.0	278
ALT (u/L)	5–40	Mean ± SD Median IQR	195.4 ± 675.3 34.0 19.0–87.0	322	98.6 ± 155.7 49.0 20.0–104.0	279
LDH (U/L)	140–280	Mean ± SD Median IQR	671.5 ± 1167.1 385.0 269.0–604.0	303	453.9 ± 332.7 379.0 256.0–517.0	265
CRP (mg/L)	<10	Mean ± SD Median IQR	150.1 ± 133.9 117.0 35.5–251.0	322	122.6 ± 139.2 90.0 46.0–173.2	287

AST: Aspartate aminotransferase; ALT: Alanine aminotransferase; LDH: Lactate dehydrogenase; CRP: C-reactive protein; SD: Standard Deviation.

**Table 3:** Clinical outcomes in subjects with a low admission plasma glutamine (<420 µmol/L) and those with a normal or elevated glutamine level (≥420 µmol/L).

Parameter	Glutamine <420 µmol/L (*n* = 193)	Glutamine ≥420 µmol/L (*n* = 137)	*p*-Value
**Disease category** (*n* (%))			
Medical	90 (57.3)	67 (42.7)	0.683; Chi^2^ = 0.16 *
Surgical	103 (59.5)	70 (40.5)	
**RVD status** (*n* = 250) (*n* (%))			
Positive	31 (68.9)	14 (31.1)	0.06; Chi^2^ = 3.36 *
Negative	111 (54.2)	94 (45.8)	
**Tuberculosis status** (*n* = 181) (*n* (%))			
Positive	8 (88.9)	1 (11.1)	**0.029; Chi^2^ = 4.75** *
Negative	94 (54.7)	78 (45.3)	
**Mechanical ventilation on admission** (*n* = 324) (*n* (%))			
Yes	145 (65.6)	76 (34.4)	**<0.001; Chi^2^ = 12.65** *
No	46 (44.7)	57 (55.3)	
**Severity of disease** (mean ± SD; median; IQR)			
APACHE II score (*n* = 327)	19.8 ± 8.9; 20.0; 12.0–26.0	16.9 ± 7.9; 16.0; 10.0–22.0	**0.003** ***
SOFA score Admission (*n* = 326)	7.6 ± 3.8; 7.0; 5.0–11.0	6.3 ± 3.6; 6.0; 3.0–8.0	**0.003** ***
SOFA score Day 7 (*n* = 291)	4.8 ± 4.2; 4.0; 1.5–7.0	4.5 ± 4.3; 3.0; 1.0–6.0	0.573 ***
**Length of stay** (days) (mean ± SD; median; IQR)			
ICU	9.5 ± 6.9; 7.0; 4.0–11.0	9.5 ± 10.7; 6.0; 4.0–11.0	0.966 **
Hospital	19.7 ± 15.5; 15.0; 8.0–28.0	17.1 ± 14.1; 13.0; 8.0–23.0	0.125 **
**Number of complications** (*n* = 305) (mean ± SD; median; IQR)	2.8 ± 3.1; 2.0; 1.0–4.0	2.5 ± 3.1; 2.0; 1.0–3.0	0.460 **
**Mortality during hospitalization** (*n* = 316) (*n* (%))			
Yes	50 (64.1)	28 (35.9)	0.305; Chi^2^ = 1.05 *
No	137 (57.6)	101 (42.4)	
**Nutrition support** (*n* (%))			
Yes	156 (62.2)	95 (37.8)	**0.016; Chi^2^ = 5.74** *
No	37 (46.8)	42 (53.2)	

Statistical analysis used: * Maximum-likelihood (ML)-Chi square test; ** *t*-test for independent samples; *** ANOVA—analysis of variance; RVD: Retroviral disease; APACHE: Acute Physiology, Age, Chronic Health Evaluation Score; SOFA: Sequential organ Failure Assessment Score; ICU: Intensive care unit AST: Aspartate aminotransferase; ALT: Alanine aminotransferase; LDH: Lactate dehydrogenase; CRP: C-reactive protein. Statistically significant values (*p* < 0.05) are indicated in bold.

**Table 4:** Biochemical outcomes (admission values) in subjects with a low admission plasma glutamine (<420 µmol/L) and those with a normal or elevated glutamine level (≥420 µmol/L).

Parameter	Unit	Baseline Data			Discharge/Day 7 data		
		Glutamine <420 µmol/L (*n* = 193)	Glutamine ≥420 µmol/L (*n* = 137)	Total (*n*) *p*-Value	Glutamine <420 µmol/L (*n* = 193)	Glutamine ≥420 µmol/L (*n* = 137)	Total (*n*) *p*-Value
Glutamine (µmol/l)	Mean ± SD	280.5 ± 93.9	724.6 ± 397.3	*n* = 330 **<0.001** *	419.1 ± 230.9	630.5 ± 772.1	*n* = 268 **<0.001** *
	Median	299.50	617.20		383.1	484.7	
	IQR	218.60–352.70	497.15–815.96		293.3–482.8	383.3–642.5	
Albumin (g/L)	Mean ± SD	24.3 ± 7.5	28.0 ± 7.5	*n* = 318 **<0.001** *	24.1 ± 17.5	26.2 ± 6.8	*n* = 280 0.208 *
	Median	24.0	29.0		23.0	26.0	
	IQR	20.0–30.0	22.0–33.0		18.0–27.0	22.0–31.0	
Urea (mmol/L)	Mean ± SD	13.9 ± 20.4	8.9 ± 7.9	*n* = 324 **0.008** *	14.1 ± 12.9	10.8 ± 12.1	*n* = 289 **0.028** *
	Median	8.0	6.4		8.4	6.9	
	IQR	5.0–16.3	3.6–11.2		4.4–21.5	4.5–11.1	
Creatinine (µmol/L)	Mean ± SD	237.4 ± 341.6	162.8 ± 187.4	*n* = 324 **0.023** *	197.9 ± 250.9	125.9 ± 157.2	*n* = 289 **0.006** *
	Median	111.0	93.0		74.0	69.5	
	IQR	70.0–250.0	62.0–141.0		56.0–229.0	54.0–107.0	
Total bilirubin (µmol/L)	Mean ± SD	20.9 ± 41.5	31.1 ± 53.7	*n* = 322 0.054 *	18.9 ± 38.8	29.0 ± 50.8	*n* = 281 0.059 *
	Median	11.0	12.0		10.0	10.0	
	IQR	7.0–20.0	6.0–27.0		6.0–18.0	6.0–22.0	
AST (u/L)	Mean ± SD	271.4 ± 1186.2	316.3 ± 981.1	*n* = 321 0.721 *	94.2 ± 154.8	70.9 ± 73.3	*n* = 278 0.132 *
	Median	61.0	44.0		46.0	52.0	
	IQR	31.0–158.0	25.0–129.5		28.5–85.0	31.0–78.0	
ALT (u/L)	Mean ± SD	144.8 ± 455.6	267.4 ± 896.9	*n*=322 0.108 *	90.8 ± 130.9	109.2 ± 184.3	*n* = 279 0.332 *
	Median	34.0	34.0		43.0	56.0	
	IQR	19.0–82.0	19.0–93.0		19.0–96.0	24.0–111.0	
LDH (u/L)	Mean ± SD	728.8 ± 1208.1	586.5 ± 1102.9	*n* = 303 0.298 *	493.1 ± 361.2	402.9 ± 284.9	*n* = 265 **0.029** *
	Median	432.0	327.5		398.5	316.0	
	IQR	293.0–648.0	244.0–546.0		290.0–573.0	230.0–491.0	
CRP (mg/L)	Mean ± SD	190.1 ± 135.9	93.3 ± 108.2	*n* = 322 **<0.001** *	144.3 ± 164.5	93.2 ± 87.1	*n* = 287 **0.002** *
	Median	172.0	53.0		114.5	72.6	
	IQR	67.0–300.0	13.0–125.0		56.0–187.0	29.9–135.9	

Statistical analysis used: * *t*-test for independent samples; AST: Aspartate aminotransferase; ALT: Alanine aminotransferase; LDH: Lactate dehydrogenase; CRP: C-reactive protein. Statistically significant values (*p* < 0.05) are indicated in bold.
